# Basement membranes at a glance

**DOI:** 10.1242/jcs.263947

**Published:** 2025-09-03

**Authors:** Rachel Lennon, David R. Sherwood

**Affiliations:** ^1^ Manchester Cell-Matrix Centre, Faculty of Biology Medicine and Health, The University of Manchester, Manchester M13 9PT, UK; ^2^ Department of Biology, Duke University, Box 90338, Durham, NC 27708, USA

**Keywords:** Basement membranes, Ageing, Disease, Membrane composition

## Abstract

Basement membranes (BMs) underlie or surround most tissues. They are formed of secreted proteins that associate with cell surfaces and are the most ancient animal extracellular matrix. Laminin and collagen IV are core components that polymerize into self-associating networks, providing BMs with an organizing scaffold and tensile strength. In humans, BMs also contain over 150 other secreted proteins, such as structural matrix components, enzymes and growth factors, as well as over 50 cell–membrane adhesion and signalling receptors. From this toolbox, BMs are tailored for tissue-specific functions, including filtration, shaping organs, connecting tissues and harbouring signals that guide cell migration and differentiation. Highlighting their importance to human health, defects in genes encoding BM proteins are associated with over 100 disease phenotypes. Advancing our understanding of BM regulation, function and dysregulation will reveal new approaches to prevent many human disorders and preserve tissue health. Here, we review our current understanding of BM composition, formation and function, and outline how BMs change with ageing and disease.

## Teaser

**Summary:** Basement membranes (BMs) are dynamic assemblies of secreted proteins uniquely tailored for diverse tissue supporting roles. Here, we review our current understanding of BM composition, formation and function, and outline how BMs change with ageing and disease.

**Figure:**
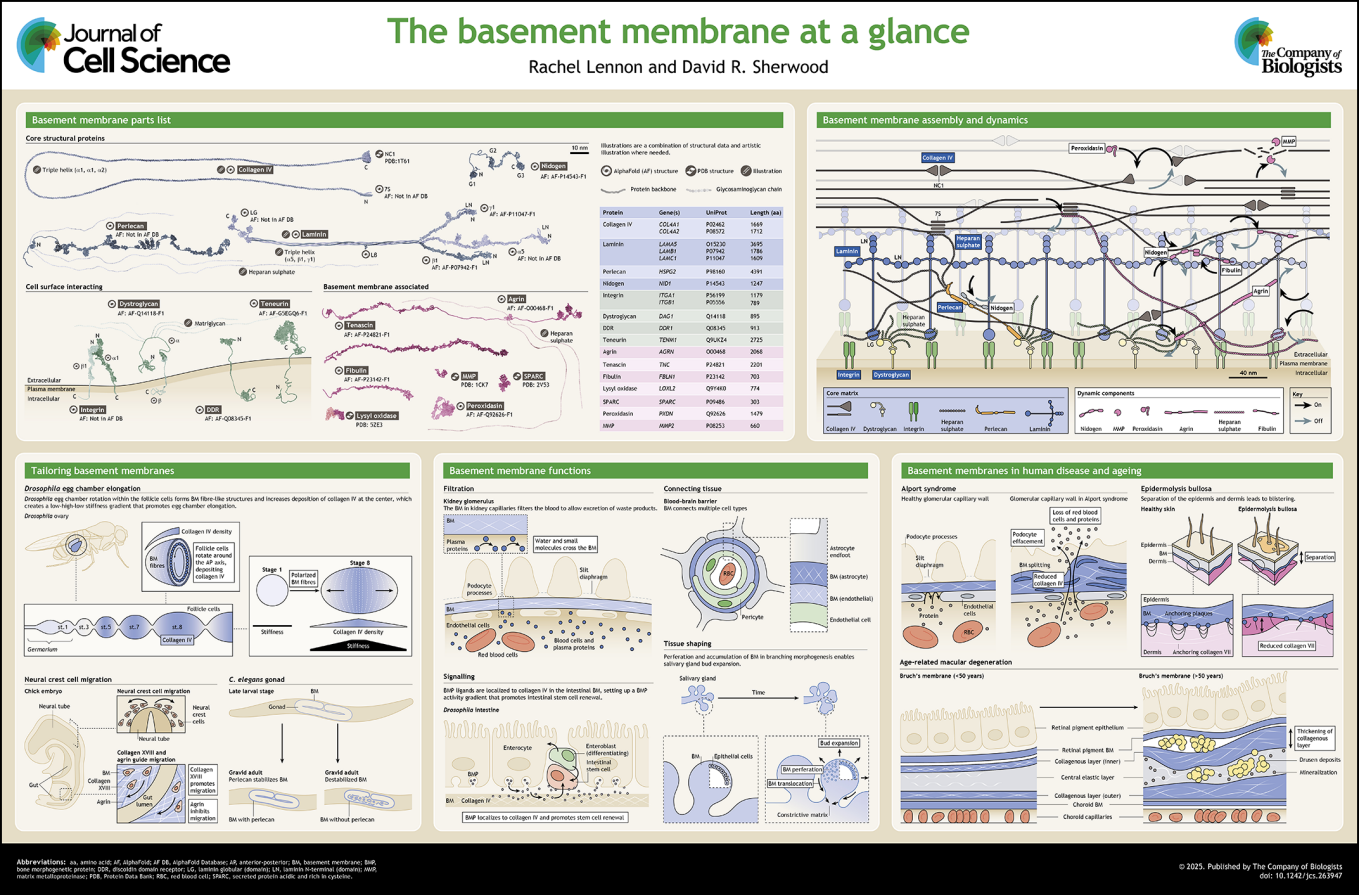
See supplementary information for a high-resolution version of the poster.

## Introduction

Basement membranes (BMs) are thin (∼100–400 nm), dense, sheet-like extracellular matrices (ECMs) that underlie or enwrap most tissues. One of the earliest extensive descriptions of BMs was by Todd and Bowman in their 1856 book ‘Physiological Anatomy and Physiology of Man’. They coined the term ‘basement membrane’ and characterized its widespread presence across tissues. Comparative studies examining early diverging metazoan lineages suggest that BMs emerged at the onset of multicellular life, as they are likely required to support complex tissue formation and function (Fahey and Degnan, 2012; Fidler et al., 2017). Corroborating this notion, BMs regulate numerous aspects of cell and tissue biology, including cell polarity, differentiation and migration, as well as tissue shape, blood filtration and resistance of tissues to mechanical forces (Pozzi et al., 2017; Naylor et al., 2021; Sherwood, 2021). Furthermore, genetic disruption or misregulation of BM components is implicated in numerous human diseases, often affecting tissue development and function (Pozzi et al., 2017). Despite their fundamental biological and clinical relevance, many aspects of BM formation, regulation and function remain unclear. Recent evidence indicates BMs are highly complex containing over 200 components in humans (Jayadev et al., 2022). The role and regulation of BM components are often challenging to dissect because of essential and frequently interlinked functions and a lack of tools to follow and manipulate individual proteins.

In this Cell Science at a Glance article and the accompanying poster, we outline our current understanding of BM composition, formation and function, and examine how BMs change with ageing and disease. We highlight recent studies and new approaches that are advancing our understanding of this crucial ECM, indicating that BMs are remarkably dynamic and tissue specific, challenging the early view of Todd and Bowman of BMs being a uniform matrix. Finally, we discuss unanswered questions in BM biology that are important to further our understanding of this highly complex and enigmatic ECM.

## A basement membrane parts list

Although BMs in different tissues have unique proteins, distinct levels and gradients of components, protein isoforms and often different receptor interactions, they consistently include four core structural molecules: laminin, collagen IV, nidogen and perlecan (Keeley et al., 2020; Jayadev et al., 2022) (see poster). These components are present across the genomes of multicellular organisms (Fidler et al., 2017; Jayadev and Sherwood, 2017) and although gene duplications have expanded gene families in vertebrates, there remains a high degree of conserved sequence homology (Hutter et al., 2000).

Laminins are glycoproteins essential for initiating BM assembly and regulating cell adhesion, polarity, differentiation and migration (Hohenester and Yurchenco, 2013; Yurchenco and Kulczyk, 2024). They are cross-shaped trimers, consisting of α, β and γ subunits associated through a long coiled-coil region. Sixteen heterotrimeric combinations have been identified from eleven subunits (α1–α5, β1–β3 and γ1–γ3) and their splice variants (Macdonald et al., 2010).

Collagen IV primarily provides tensile strength and structural support to BMs (Summers et al., 2023). Its protomers consist of three α-chains that intertwine to form a triple-helix structure. These protomers form networks within BMs through covalent cross-links that connect two interacting C-terminal NC1 domains and four N-terminal 7S domains. The network is further strengthened through lateral twisting interactions between type IV collagen protomers (Timpl et al., 1981; Yurchenco and Ruben, 1987). Mammals encode six α-chains (α1–α6), forming at least three distinct collagen IV trimers, denoted by α followed by the three numbers. The α112 network is ubiquitous, whereas the α345 and α556 networks have restricted tissue localization and are expanded members of the collagen IV family that arose in early vertebrates (Pokidysheva et al., 2023).

Laminin and collagen IV networks connect through the crosslinking proteins nidogen, perlecan and agrin (Hohenester and Yurchenco, 2013). The networks might also associate through direct laminin–collagen IV interactions (Charonis et al., 1985; Yurchenco and Kulczyk, 2024). Nidogen is a glycoprotein that stabilizes BMs through direct binding to both laminin and collagen IV (Aumailley et al., 1993; Jayadev et al., 2022), and also through high-affinity binding of the nidogen G2 domain to the immunoglobulin-like domain 3 of perlecan (Kvansakul et al., 2001). Perlecan and agrin are heparan sulphate proteoglycans (HSPGs) that further connect the laminin and collagen IV networks and stabilize laminin interactions with the cell surface (Behrens et al., 2012; Hohenester and Yurchenco, 2013). Perlecan, agrin and other BM-associated HSPGs have negatively charged heparan sulphate sugar residues, retaining water, contributing to BM hydration and protection from osmotic stress (Halfter et al., 2015; Töpfer et al., 2022). HSPGs also bind growth factors, activating diverse signalling pathways that regulate cell proliferation, migration and differentiation (Yurchenco, 2011; Gubbiotti et al., 2017).

In addition to core components, numerous additional proteins associate with BMs (Yurchenco, 2011; Randles et al., 2017; Jayadev et al., 2022). These include cell surface interactors such as integrins, dystroglycans and tyrosine kinase discoidin domain receptors (DDRs) (Borza et al., 2015), which bind core BM ligands and mediate cell adhesion and signalling. Other receptors, such as TGFβ and Robo receptors, are activated by TGFβ and Slit ligands, respectively, within BMs (Jayadev et al., 2022). The teneurin receptor, which is essential for brain development and synaptic organization, is also associated with BMs and has poorly understood roles in BM regulation (Tucker and Chiquet-Ehrismann, 2006; Topf and Drabikowski, 2019). BMs also contain secreted regulatory proteins, including proteases [e.g. matrix metalloproteinases (MMPs), ADAMs (a disintegrin and MMP) and their inhibitors], which facilitate matrix degradation and generation of biologically active fragments (matrikines) (Bonnans et al., 2014; Apte and Parks 2015; Page-McCaw 2008; Okada et al., 2017; Eckersley et al., 2023); cross-linking enzymes (e.g. lysyl oxidase and peroxidasin), which strengthen collagen IV networks and enhance BM integrity (López-Jiménez et al., 2017); and matricellular proteins [e.g. fibulins, hemicentins, secreted protein acidic and rich in cysteine (SPARC), tenascin], which modulate matrix assembly and cell interactions (Raja et al., 2023). Collagen VII, also commonly found in vertebrate BMs, forms anchoring fibrils that tether epithelial BMs to underlying connective tissue (Watanabe et al., 2018).

The molecular mass of BM proteins can vary significantly, with many being extremely large. This likely bestows structural integrity and provides multiple interaction sites for binding various molecules, enabling the organization of complex scaffolds. For example, the non-structural matricellular protein SPARC is ∼40 kDa (Kuhn and Mason, 1995), whereas laminin trimers range from 400–900 kDa (up to 110 nm long from their C-terminal LG domains to their N-terminal LN domain tips), and the collagen IV trimer is ∼540 kDa (400 nm long) (Timpl et al., 1981; Leblond and Inoue, 1989; Hohenester, 2019). These trimers are individual units within extensive polymer networks that likely reach megadalton-scale mass. Additionally, post-translational modifications (PTMs), such as N- and O-linked glycosylation and extensive glycosaminoglycan chains, further increase protein mass and functional diversity (Gubbiotti et al., 2017; Fidler et al., 2018; He et al., 2024).

A comprehensive parts list of BM components was recently assembled using bioinformatics and fluorescent tagging in *Caenorhabditis elegans*. This study identified 160 human matrix proteins and 62 cell surface interactors localized to the BM zone, with orthologues in mice, zebrafish, *Drosophila* and *C. elegans* (Jayadev et al., 2022). Many gene families have expanded in vertebrates, suggesting increased BM complexity. Given that numerous matrix proteins, particularly the regulatory factors, are present at low levels, some BM components might remain cryptic. Nevertheless, this resource allowed deep bioinformatic approaches that implicated several matrix proteins, such as papilin and perlecan, as key regulators in the formation and function of BMs (Jayadev et al., 2022). To organise and disseminate information about BMs, the online database basementmembraneBASE (https://bmbase.manchester.ac.uk/) was created alongside that study (Jayadev et al., 2022).

## BM assembly and dynamics

Studies in mice, *Drosophila* and *C. elegans* have revealed that laminins are essential for initiating BM assembly (Smyth et al., 1999; Huang et al., 2003; Matsubayashi et al., 2017). Laminin polymerization starts at the cell surface, where C-terminal LG domains bind to integrin and dystroglycan cell adhesion receptors, as well as sulphated glycolipids (see poster). Meanwhile, the N-terminal LN domains of α-, β- and γ-subunits form non-covalent interactions with neighbouring laminin LN domains (Yurchenco and Kulczyk, 2024). Engagement with integrin and dystroglycan receptors also connects BMs to the cytoskeleton through the intracellular domains of these adhesion receptors and facilitates cell signalling (described below and see poster).

Following laminin network assembly, other core BM proteins are recruited. This process is best understood in *C. elegans*, where all core BM components and most accessory BM proteins have been endogenously tagged with the fluorophore mNeonGreen (Keeley et al., 2020; Jayadev et al., 2022). Laminin, nidogen and papilin are the first detectable BM components in tissues and appear at the end of gastrulation, suggesting that laminin recruits nidogen and papilin during its assembly. Papilin, a highly conserved and abundant BM component, is ubiquitous in *C. elegans* BMs (Keeley et al., 2020) and broadly expressed in vertebrates, suggesting that it might be an overlooked core component (Jayadev et al., 2022). Although its biochemical interactions remain uncharacterized, papilin is composed of many different structural domains that are found separately in BM proteins, including the netrin receptor DCC, ADAMTS proteases, peroxidasins and proteoglycans. The assembly of these different and diverse domains within papilin might be required for its crucial function in promoting type IV collagen BM turnover (Keeley et al., 2020; Jayadev et al., 2022). By the end of gastrulation, the *C. elegans* laminin-binding integrin receptor and the sole dystroglycan receptor are present on all cell surfaces that contain BMs. At ∼100 min later, collagen IV and its crosslinking enzyme peroxidasin appear in BMs, followed 60 min later by perlecan. Other matricellular proteins, including SPARC, collagen XVIII and spondin, as well as the *C. elegans* collagen receptor DDR, also appear in BMs at this later time. A similar order of assembly has been observed in *Drosophila*, where fluorescently tagged laminin, nidogen, collagen IV and perlecan display sequential recruitment (Matsubayashi et al., 2017, 2020). In mutant mice lacking collagen IV, laminin and nidogen still form a BM-like matrix (Pöschl et al., 2004), suggesting the same recruitment order. Interestingly, although collagen IV assembly depends on laminin during BM formation in embryogenesis, a distinct laminin-independent or significantly reduced laminin-dependent mode(s) of collagen IV assembly occurs later in specific contexts, like in the *C. elegans* larval pharyngeal BM, and during *Drosophila* epidermal wound repair and *Drosophila* egg chamber development (Ramos-Lewis, LaFever and Page-McCaw, 2018; Jayadev et al., 2019; Töpfer et al., 2022), indicating tissue-specific mechanisms of BM assembly. Emerging studies using photobleaching, pulse-chase experiments and photoconversion of fluorescently tagged BM components (Box 1), along with time course proteomics analysis (Box 2), have revealed that BMs, once assembled, are remarkably dynamic structures.

Box 1. Imaging basement membrane dynamics Investigations using BM components endogenously tagged with genetically encoded fluorescent proteins are yielding surprising insights into BM dynamics (Keeley et al., 2020; Matsubayashi et al., 2020; Stramer and Sherwood, 2024). Fluorescence recovery after photobleaching (FRAP) of core and BM-associated proteins in *C. elegans* has shown that collagen IV and laminin have relatively stable BM associations (half-life recovery of hours). In contrast, fibulin, nidogen and agrin, have half-life recovery within ∼15 min, primarily through lateral movement within the BM (Keeley et al., 2020). Although the significance of this rapid lateral movement is unclear, it might allow BMs to dynamically respond to mechanical changes in tissues. Furthermore, as mobile matrix components, such as agrin, bind growth factors, BMs might serve as molecular highways for growth factor trafficking. Pulse-chase experiments with fluorescently tagged collagen IV during *Drosophila* embryogenesis have revealed a 14-h degradation half-life. Interestingly, experiments examining a photoconvertible collagen IV in *Drosophila*, have shown a 4-h association half-life with the BM, suggesting that collagen IV, even though it can be cross-linked in networks, associates and dissociates from the BM multiple times before degradation (Matsubayashi et al., 2020). Recently developed mouse models with fluorophore-tagged laminin and collagen IV (Morgner et al., 2023; Jones et al., 2024; Wuergezhen et al., 2025) show an ∼3-h replacement half-life for collagen IV in the developing hair follicle BM, which is similar to the turnover seen in *Drosophila* and *C. elegan*s (Keeley et al., 2020; Matsubayashi et al., 2020). Together, these observations indicate that BMs are dynamic during tissue growth and differentiation.

Box 2. Proteomic analysis of basement membrane dynamics Proteomic studies provide insights into protein dynamics through time-course measurements of protein abundance or labelled proteomics, which can reveal both abundance and protein half-lives. Although these approaches lack spatial resolution, they divulge the dynamics of multiple proteins within a single analysis.

Proteomic approaches to studying BM dynamics remain limited, with the kidney being the most extensively researched. In both mouse and human kidneys, ageing is associated with reduced levels of core BM components and increased deposition of interstitial matrix proteins like fibrillar collagens (Randles et al., 2021). This compositional change is also seen in various kidney diseases, suggesting a common injury response (Randles et al., 2021). To measure BM protein turnover in the kidney, a labelled proteomics workflow was developed in which mice were fed exclusively isotopic nitrogen-15 (in contrast to normal nitrogen-14) from 3 weeks of age for 12 weeks before harvesting the kidney (Liu et al, 2020). Analysis identified a pool of long-lived BM components (containing a high percentage of nitrogen-14), including collagen IV, collagen VI, laminin and perlecan, with half-lives of ∼12 weeks or more. Notably, at ∼12 weeks, collagen IV in the adult kidney is over two orders of magnitude more long-lived than the ∼14-h turnover observed in *Drosophila* embryos. Not all BM components were equally stable; for example, only 5% of nitrogen-14 remained in agrin, and integrin showed no detectable nitrogen-14 after 12 weeks (Liu et al, 2020). This study suggests that core BM components have a much slower turnover rate in adult BMs compared to during development, when tissues are growing and undergoing morphogenetic shape changes.

## Tailoring basement membranes

Although tissue-specific BMs have distinct compositions, the significance of these differences, and how they are established, is only beginning to emerge (Sherwood, 2021; Díaz-de-la-Loza and Stramer, 2024). One of the most interesting examples is the *Drosophila* egg chamber, where a low-high-low collagen IV stiffness gradient forms along the anterior-posterior axis (see poster). This gradient is shaped by the egg chamber epithelial cell layer, which undergoes collective cell migration and rotation within the encasing BM during stages 1–8 of egg chamber development. As the epithelial cells rotate, they secrete collagen IV into the planar BM matrix and the rotations help shape collagen IV into perpendicular fibrils. Together, the planar BM matrix and embedded fibrils form the stiffness gradient (Isabella and Horne-Badovinac, 2016; Crest et al., 2017). This gradient then instructs epithelial cell orientation and shape changes that elongate egg chamber growth (Chen et al., 2019; Cerqueira Campos et al., 2020; Sherwood, 2021).

Dynamic tailoring of BM composition also provides directional cues for cell migration and neuronal processes (Sherwood, 2021). An example is the vagal neural crest cells (see poster), which undergo a long migration along the BM of the intestinal blood vessel to populate the entire gut. During early migration, the neural crest cells secrete collagen XVIII into the BM, promoting their movement. Later, they switch to secreting agrin, which halts migration, allowing them to settle and form the enteric nervous system (Nagy et al., 2018).

BM compositional changes throughout a tissue can also support transitions in tissue needs. In *C. elegans*, the BM-enwrapped gonad adapts as the organ increases more than 90-fold in volume, from its initial formation at the L1 larval stage to the completion of growth in early adulthood (see poster; Jayadev et al., 2019). Core BM components, such as laminin, collagen IV and nidogen, are present throughout this growth, but perlecan is initially absent. It is later recruited in the early adult stage through an unknown mechanism, where it maintains BM integrity and supports gonadal tissue (Jayadev et al., 2022). Perlecan might be specifically required in the adult to help the BM withstand contractions of gonadal myoepithelial cells that drive oocyte ovulation (Kelley and Cram, 2019).

## BM functions

BMs are highly complex and play multiple roles in supporting tissues. They are an order of magnitude stiffer than epithelial cell layers, and one of their primary functions is to provide mechanical support (Halfter et al., 2015). As a result of this crucial role, the loss or genetic defects in core BM components leads to embryonic lethality and disruption of diverse tissues throughout animal life (Huang et al., 2003; Pöschl et al., 2004; Mao et al., 2015; Yurchenco and Kulczyk, 2024).

Beyond mechanical support, BMs function as selective barriers that regulate the passage of molecules and cells between tissue compartments. The clearest example is the glomerular BM in the kidney (see poster), which functions as a filtration barrier, blocking large proteins and blood cells while allowing water and small solutes to pass (Preston et al., 2025). BMs are also found at several other barriers, including Reissner's membrane in the cochlea, the blood–brain barrier, the lung alveoli, the blood–testes barrier and at Bruch's membrane in the eye; in these cases, BMs might directly mediate selective filtration of diverse molecules (Zdebik et al., 2009; Cheng and Mruk, 2012; Keeley and Sherwood, 2019).

At tissue barriers, BMs also serve as anchoring sites for tissue–tissue connections. For example, at the blood–brain barrier (see poster), the BM from the astrocyte feet and endothelial cells attach (a BM–BM connection), linking the tissues (Xu et al., 2019). Similar BM–BM connections also occur where muscles connect with tendons at myotendinous junctions (Welcker et al., 2021) and during morphogenetic processes, including mouth formation, somite positioning and optic fissure closure (Keeley and Sherwood, 2019). Despite the prevalence and importance of these BM–BM connections, their composition and construction remain poorly understood. Work in *C. elegans* has uncovered a BM–BM matrix adhesion system between uterine and epidermal tissues, which relies on integrin and DDR matrix-binding receptors, along with the matricellular proteins hemicentin, fibulin-1 and collagen IV, which link the juxtaposed BMs (Morrissey et al., 2014; Gianakas et al., 2023; Park et al., 2023). Intriguingly, loss of these components disrupts tissue connections in vertebrates, suggesting a conserved adhesion system (Feitosa et al., 2012; Keeley and Sherwood, 2019; Welcker et al., 2021).

BMs also act as signalling platforms that instruct morphogenetic cellular behaviour, cell survival, proliferation and differentiation. Core components like laminins signal through integrin and dystroglycan receptors to mediate epithelial polarization (Matlin et al., 2017), whereas collagen IV signals through DDR1 to regulate proliferation, differentiation and cell–matrix adhesion (Leitinger, 2014; Park et al., 2023). BM–integrin engagement can also promote signalling through other pathways, such as EGF receptor activation (Yurchenco, 2011; Elbediwy et al., 2016). Many BM components are cleaved by proteases, releasing matrikines, such as endostatin cleaved from collagen XVIII (Okada et al., 2017). Furthermore, heparan sulphate chains on HSPGs, and the collagen IV and perlecan proteins themselves, bind growth factors and cytokines, activating cell signalling pathways and influencing cell behaviour (Wang et al., 2008; Yurchenco, 2011). For example, in the *Drosophila* midgut, enterocytes secrete bone morphogenetic protein (BMP) basally, where it binds to collagen IV in the underlying BM, concentrating BMP and activating BMP signalling in intestinal stem cells, thereby promoting their self-renewal (see poster; Tian and Jiang, 2014).

Another BM role is in sculpting tissue architecture. BM properties affect tissue shape by directing specific cellular behaviours. The best characterized example is outlined above, where collagen IV fibrils and a collagen stiffness gradient patterns epithelial F-actin stress fibres (collagen fibrils) and directs cell reorientation (stiffness gradient) driving egg chamber elongation (Chen et al., 2019; Cerqueira Campos et al., 2020). Other examples are emerging, although how physical BM properties translate to cellular behaviour that mediate tissue shape changes remains unclear. In the vertebrate salivary gland, MMP-generated perforations at the bud tip facilitates BM movement (whole BM sheet translocation) away from the tip, concentrating BM around the duct to promote outgrowth (see poster; Harunaga et al., 2014). Similar BM remodelling occurs in the lung, mammary gland and kidney (Harunaga et al., 2014; Spurlin et al., 2019) and, in the early mouse epiblast, BM perforations generated by MMPs promote primitive streak extension and gastrulation (Kyprianou et al., 2020). Tissue shape is also influenced by the balance of BM components. For example, perlecan counteracts the contraction of collagen IV, helping define the shapes of the *Drosophila* wing disc, salivary gland and ventral nerve cord (Pastor-Pareja and Xu, 2011). Finally, in a fascinating illustration of BM shaping tissues, *de novo* collagen IV polymerization appears to drive ventral nerve cord condensation in *Drosophila* (Serna-Morales et al., 2023).

## BMs in human disease and ageing

Human genetic conditions caused by defects in BM genes highlight the essential role of BM proteins in maintaining tissue integrity. Approximately 220 genes encode BM ligands or receptors, and defects in these genes are associated with ∼100 distinct human phenotypes (Jayadev et al., 2022). For example, Alport syndrome (see poster) is a multisystem genetic disorder caused by variants in the collagen IV genes *COL4A3*, *COL4A4* and *COL4A5*. These variants affect a tissue-restricted isoform of collagen IV, and result in abnormal kidney filtration, hearing loss and eye abnormalities (Puapatanakul and Miner, 2024). Over 3500 pathogenic or likely pathogenic variants in these Alport genes are reported in the genetic variant database ClinVar (https://www.ncbi.nlm.nih.gov/clinvar/; Landrum et al., 2018). Loss-of-function variants are typically associated with severe clinical phenotypes (Savige et al., 2022), whereas missense variants, which predominantly involve replacement of glycine with another amino acid, result in milder phenotypes, but the variants do disrupt the integrity of the collagen IV triple helix, compromising its structural stability (Yeo et al., 2020). Population genomic studies indicate that variants in the Alport genes might be present in up to one in ∼100 individuals, suggesting a broader spectrum of phenotypes than traditionally recognized (Gibson et al., 2021). Another multisystem genetic disorder involving collagen IV is Gould syndrome, caused by variants in *COL4A1* and *COL4A2*. These variants affect the α112 collagen IV isoform and lead to a range of clinical phenotypes, including stroke, eye defects, muscle and kidney problems (Jeanne and Gould, 2017). Together, Alport and Gould syndromes highlight the important role of collagen IV in BM function.

Epidermolysis bullosa (EB) is another genetic disorder caused by defects in several BM-associated genes (see poster). *LAMA3*, *LAMB3* and *LAMC2* encode laminin-332, a network essential for skin integrity; defects in this laminin network result in junctional EB (Danescu et al., 2024). The hemidesmosome-anchoring protein collagen VII, encoded by *COL7A1*, is another crucial BM-anchoring protein that links the epidermal BM to the underlying dermal ECM, and variants cause dystrophic EB (Nyström, 2025). Individuals with EB experience chronic pain, scarring and increased infections from fragile skin that is highly susceptible to blistering, even from minor trauma or friction. Although treatments for EB have been mainly focussed on symptom management, recent advances in cell and gene therapy over the last 5 years offer hope that transformative treatments might soon be possible for individuals living with EB (Hirsch et al., 2017; Nyström, 2025).

In addition to genetic disorders, ageing impacts BM function. This is well-documented in the retina, where changes in BM structure lead to age-related macular degeneration (AMD), a major cause of visual impairment affecting 196 million people worldwide (Fleckenstein et al., 2024). The retinal pigment epithelium interacts with Bruch's membrane, a specialized five-layered ECM structure essential for retinal function (Clark et al., 2025). Oxidative stress and proteases such as high-temperature requirement protein A1 (HTRA1) and MMPs alter the composition of Bruch's membrane in early stages of AMD, disrupting the function of the retinal cells leading to visual impairment (Booij et al., 2010). A hallmark of AMD is the buildup of lipid deposits, known as drusen, within the Bruch's membrane at the junction of ECMs derived from neuroectodermal and mesodermal origins (Booij et al., 2010). This junction is a BM–BM tissue connection, and it is interesting that variants in the hemicentin gene *HMCN1* (a protein known to mediate BM linkage in *C. elegans*) have been associated with AMD (Thompson et al., 2007; Morrissey et al., 2014). Alongside the compositional changes that occur in Bruch's membrane in AMD, immune dysregulation plays a central role in AMD. Variants in complement factor H are a genetic risk factor, and complement-blocking therapies are being used in the clinic (Toomey et al., 2018; de Jong et al., 2023). Further understanding of the role of BM components in tissue connections in the retina and in regulating immune responses might enable the development of more effective therapies for AMD.

## Conclusions and future perspectives

BMs are not uniform static structures as originally postulated, but instead are diverse and dynamic. These specialized ECMs have been integral partners to animal tissues for hundreds of millions of years, and we are likely vastly underestimating their complexity and regulation. Given the large number of distinct and interlinked components, it is important to continue using conditional and targeted loss of BM components in animal models to understand precise functions of individual BM components, rather than relying solely on analysis of indirect downstream effects. Furthermore, cell–BM interactions are dynamic and reciprocal. Advancing live imaging technologies to visualize back-and-forth interactions between cellular processes and matrix components will be crucial to understanding this communication. BMs bear mechanical loads and provide core structural tissue support. But which BM components bear these loads, when and how? To answer this, we must develop molecular force sensors within BM components for *in vivo* studies to begin to understand the role of mechanics in BM regulation and function. Although existing literature portrays BMs as largely stiff, immobile structures, this cannot be the case. BM-encased tissues routinely stretch and recover their form. Yet, few studies have addressed how BMs are elastic. Although *Drosophila* and *C. elegans* models have advanced our understanding of BM dynamics in development, BM regulation during adult homeostasis and ageing remains poorly understood. These powerful invertebrate models are poised to explore these gaps. In vertebrates, endogenous tagging of BM components will be crucial for understanding BM behaviour in tissue-specific contexts, disease models and regenerative processes. A deeper understanding of BM regulation also offers an opportunity to identify new diagnostic and therapeutic strategies that might detect and restore BM function. Overall, a multidisciplinary approach, integrating biology, biomechanics and systems biology, will be vital to fully unravel the complexity of the BM and to develop innovative interventions to combat BM-related diseases.

## Poster

Poster

## Panel 1. Basement membrane parts list (I)

Panel 1. Basement membrane parts list (I)

## Panel 2. Basement membrane parts list (II)

Panel 2. Basement membrane parts list (II)

## Panel 3. Basement membrane assembly and dynamics

Panel 3. Basement membrane assembly and dynamics

## Panel 4. Tailoring basement membranes

Panel 4. Tailoring basement membranes

## Panel 5. Basement membrane functions

Panel 5. Basement membrane functions

## Panel 6. Basement membranes in human disease and ageing

Panel 6. Basement membranes in human disease and ageing
