# Supplementary figures and images for: Basement membranes at a glance
Source: J Cell Sci. 2025 Sep 3;138(17):jcs263947. doi: 10.1242/jcs.263947 (PMC12450471; doi:10.1242/jcs.263947)

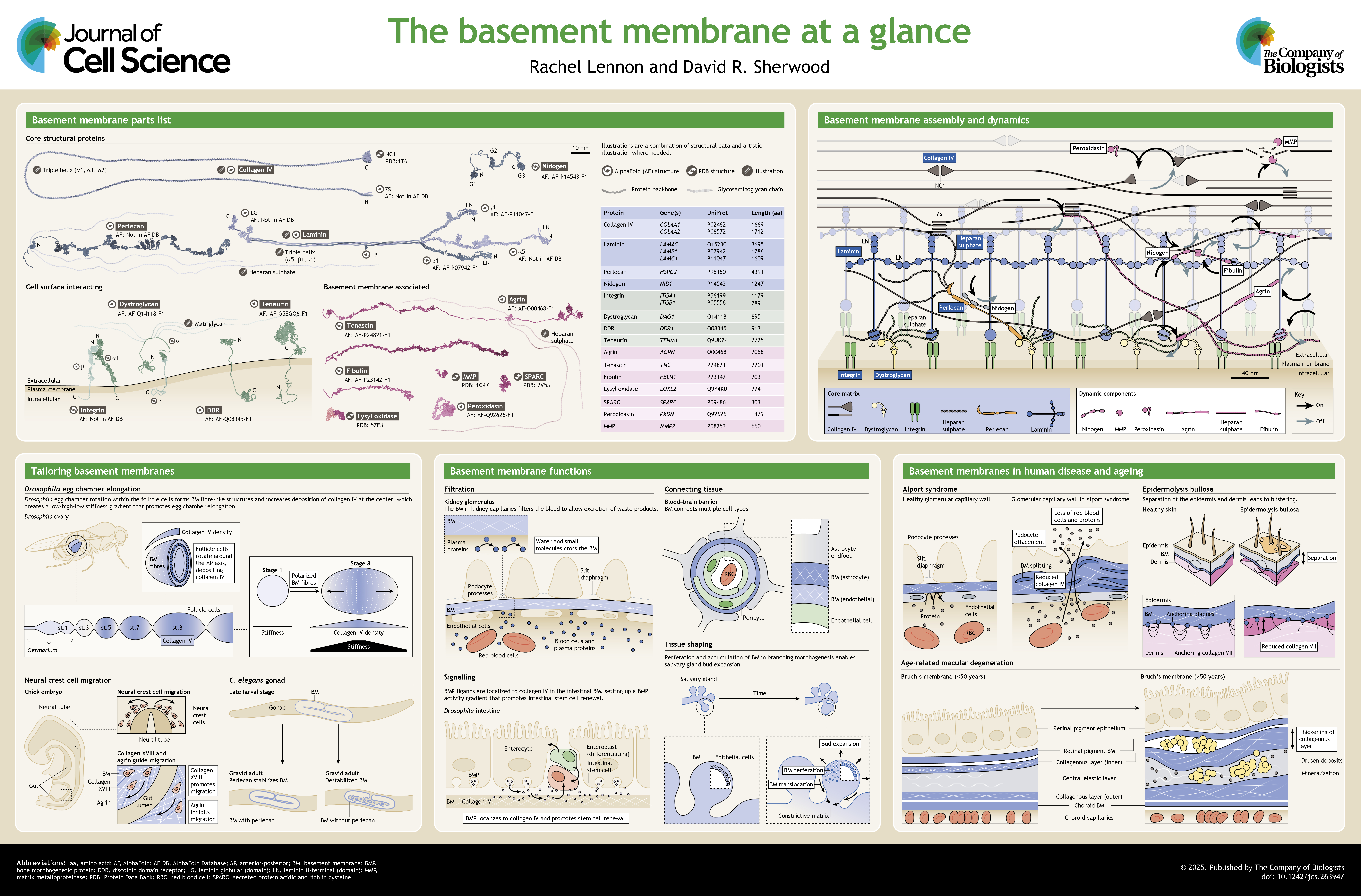

Supplement: Poster [file joces-138-263947-s1.jpg]

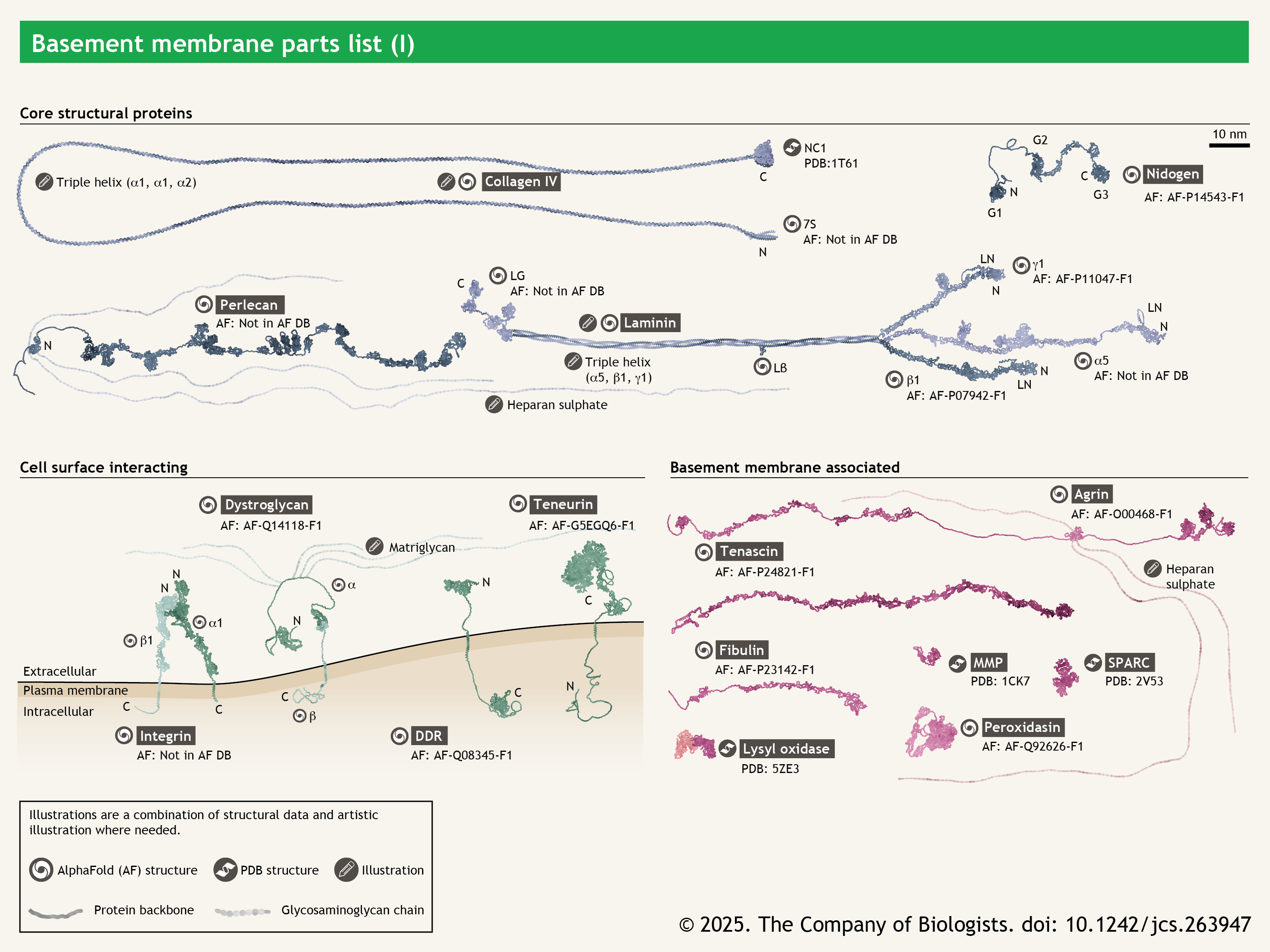

Supplement: Panel 1. Basement membrane parts list (I) [file joces-138-263947-s2.jpg]

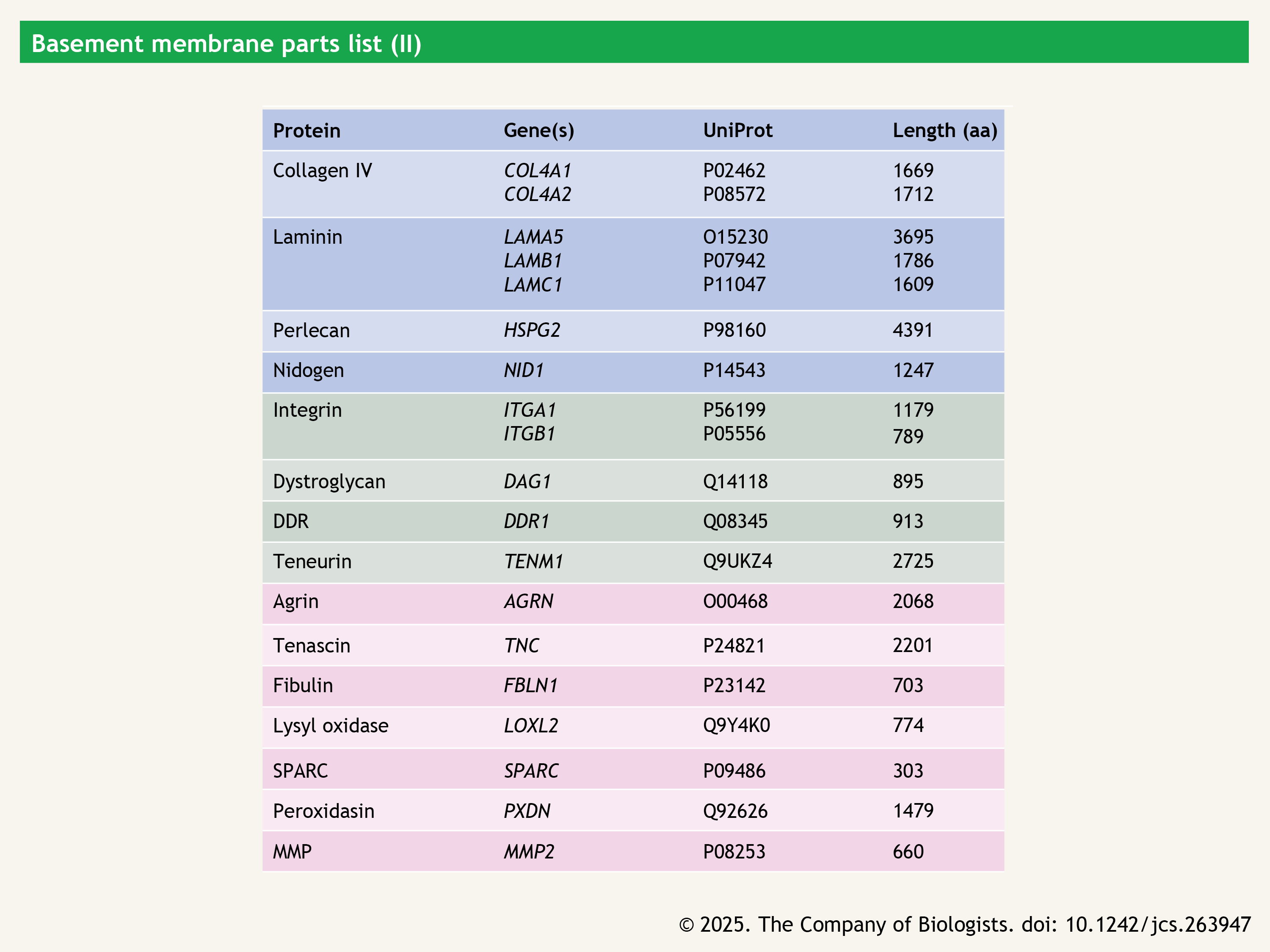

Supplement: Panel 2. Basement membrane parts list (II) [file joces-138-263947-s3.jpg]

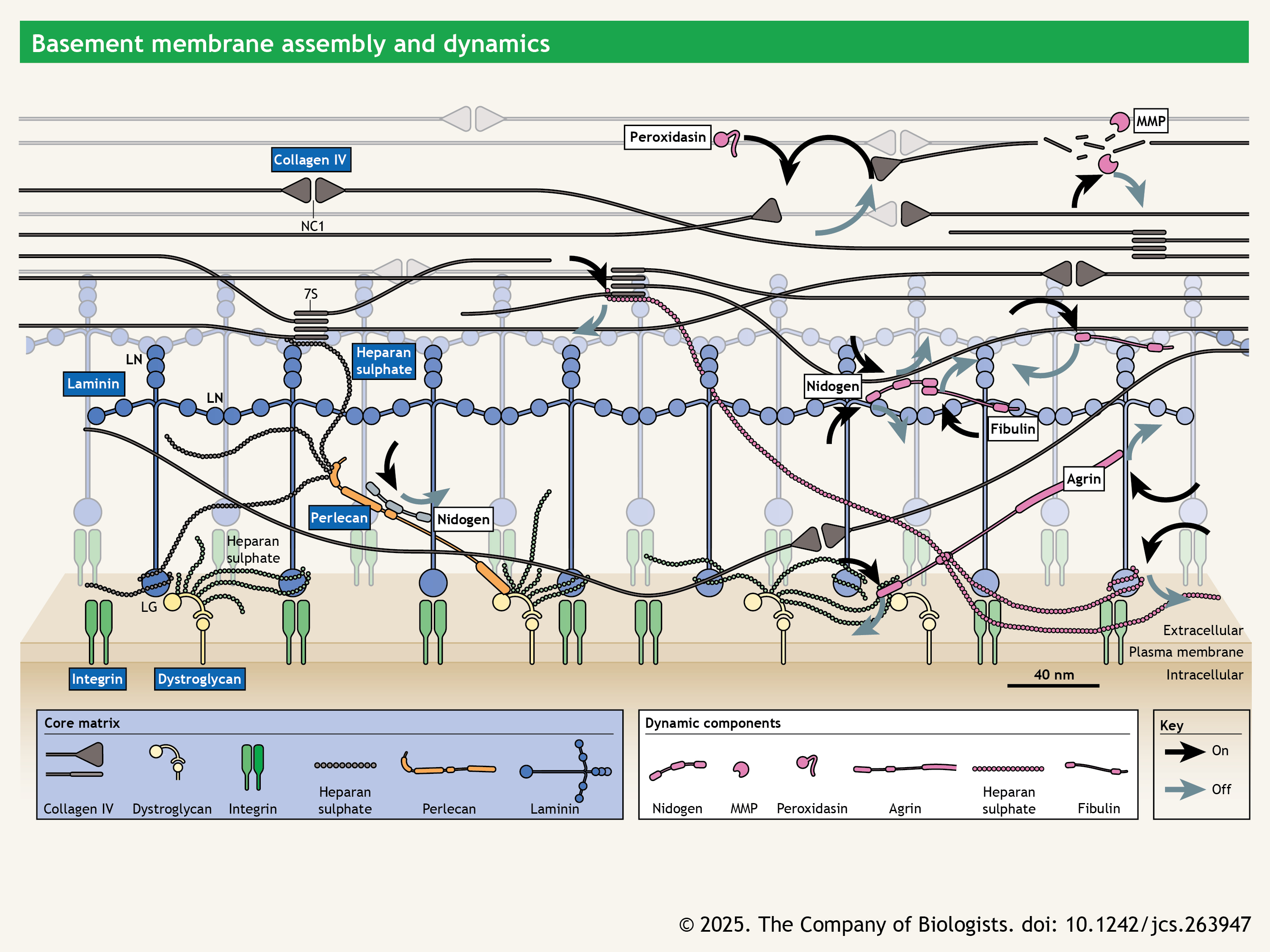

Supplement: Panel 3. Basement membrane assembly and dynamics [file joces-138-263947-s4.jpg]

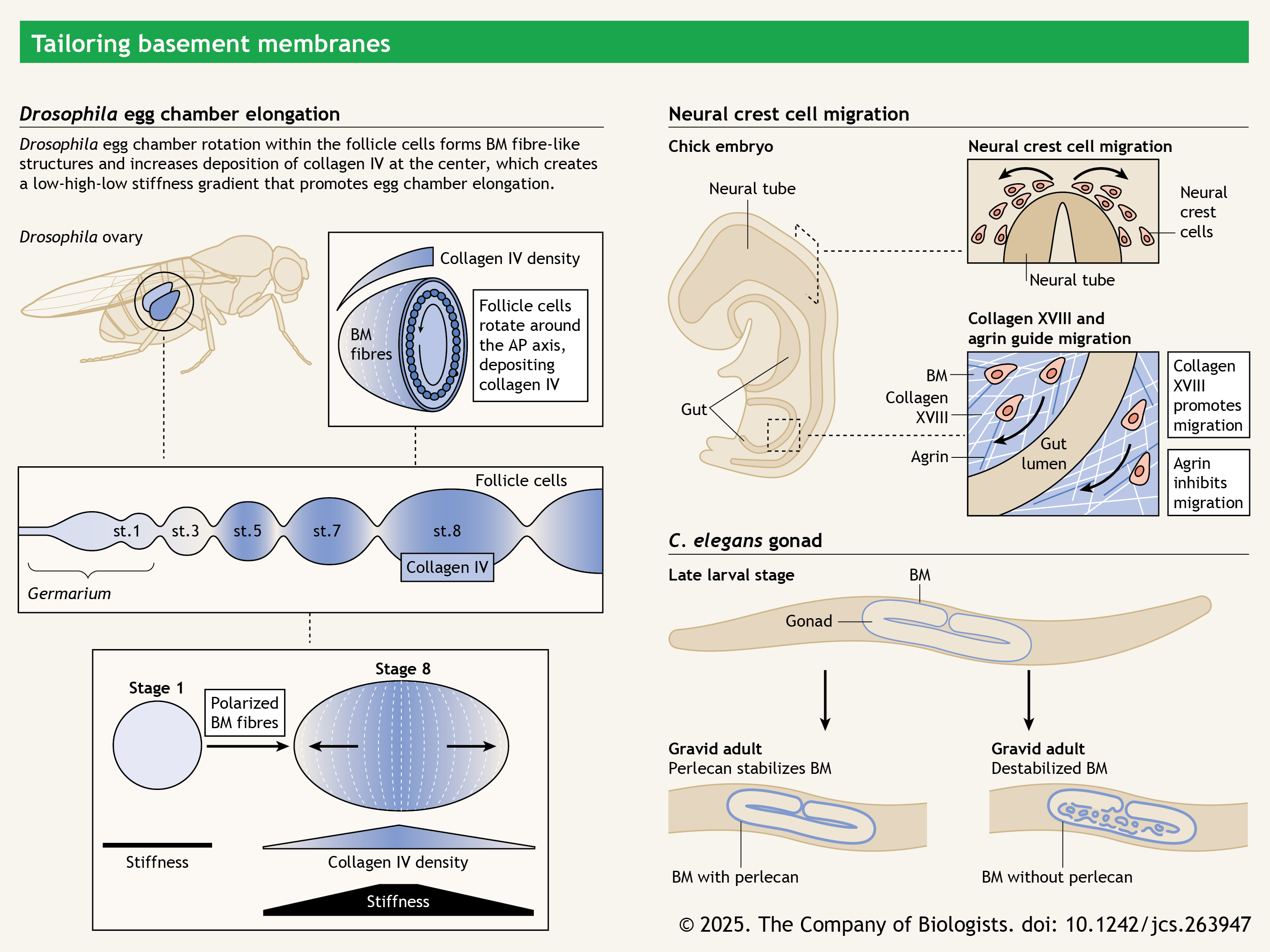

Supplement: Panel 4. Tailoring basement membranes [file joces-138-263947-s5.jpg]

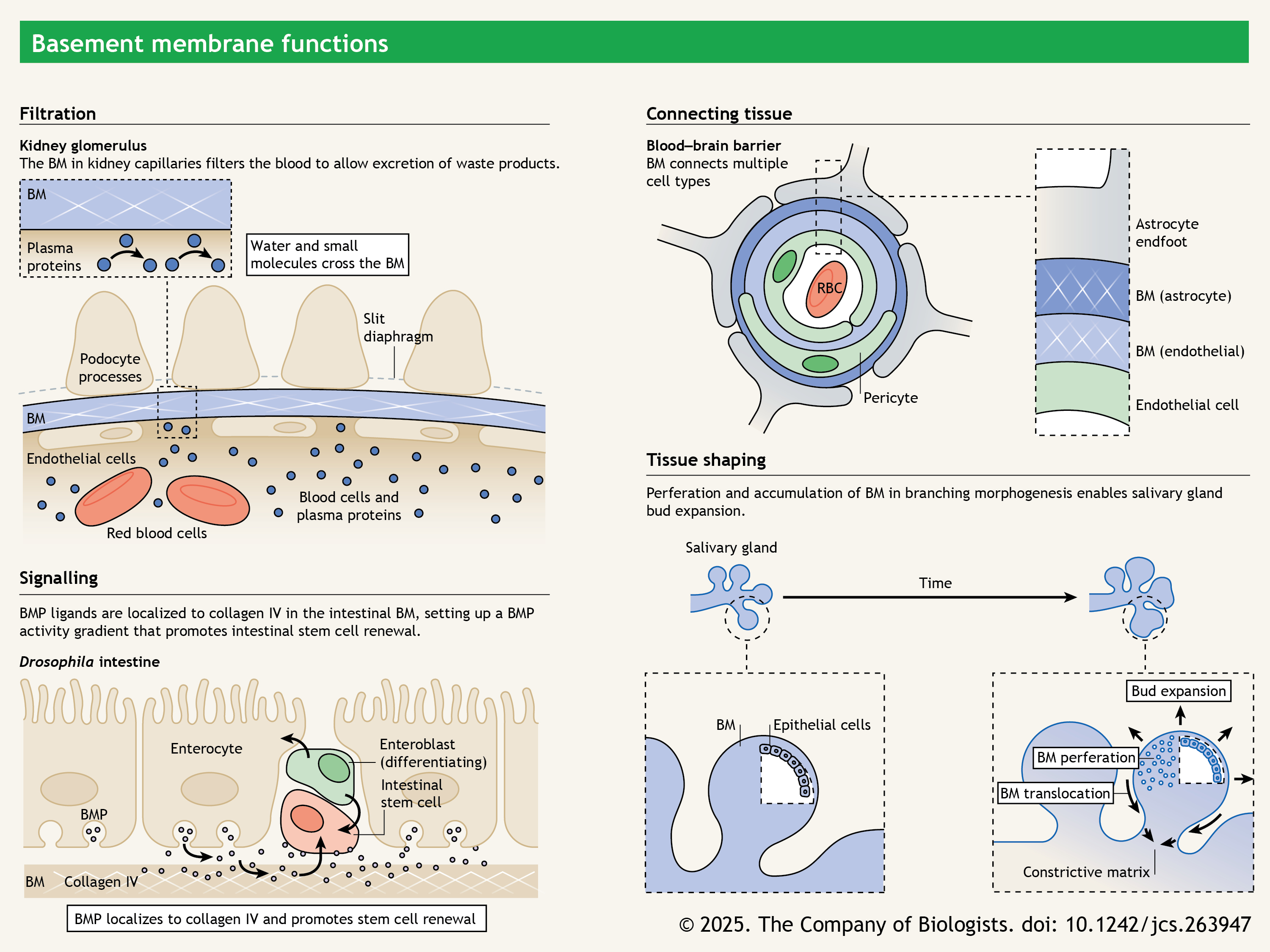

Supplement: Panel 5. Basement membrane functions [file joces-138-263947-s6.jpg]

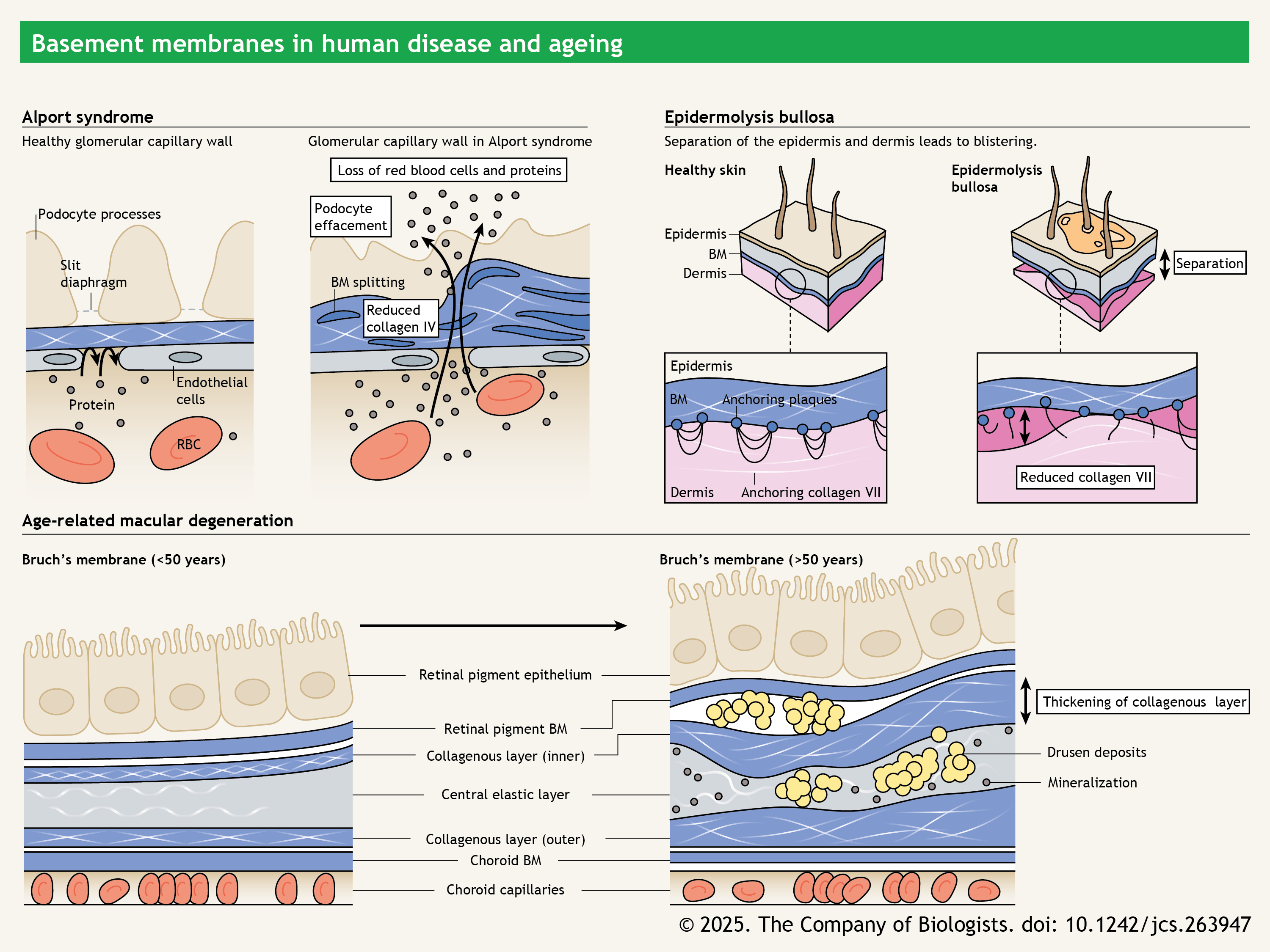

Supplement: Panel 6. Basement membranes in human disease and ageing [file joces-138-263947-s7.jpg]
